# Photocatalytic Degradation of Methyl Orange over Metalloporphyrins Supported on TiO_2_ Degussa P25

**DOI:** 10.3390/molecules17021149

**Published:** 2012-01-25

**Authors:** Xian-Tai Zhou, Hong-Bing Ji, Xing-Jiao Huang

**Affiliations:** School of Chemistry and Chemical Engineering, Key Laboratory of Low-Carbon Chemistry & Energy Conservation of Guangdong Province, Sun Yat-sen University, 510275, Guangzhou, China

**Keywords:** metalloporphyrins, photocatalytic, methyl orange, degradation

## Abstract

The photocatalytic activity of *meso*-tetraphenylporphyrins with different metal centers (Fe, Co, Mn and Cu) adsorbed on TiO_2_ (Degussa P25) surface has been investigated by carrying out the photodegradation of methyl orange (MO) under visible and ultraviolet light irradiation. The photocatalysts were characterized by X-ray diffraction (XRD), scanning electron microscopy (SEM), diffuse reflectance UV (DRS-UV-vis) and infrared spectra. Copper porphyrin-sensitized TiO_2_ photocatalyst (CuP-TiO_2_) showed excellent activity for the photodegradation of MO whether under visible or ultraviolet light irradiation. Natural Bond Orbital (NBO) charges analysis showed that methyl orange ion is adsorbed easier by CuP-TiO_2_ catalyst due to the increase of induced interactions.

## 1. Introduction

Due to its nontoxicity, stability, high photocatalytic activity and recyclability the photocatalytic degradation of pollutants on the surface of titanium dioxide (TiO_2_) has been intensively studied as a way to solve environmental problems [1,2,3]. Despite the many known advantages of using TiO_2_, it suffers from the shortcoming of having a large band gap (~3.2 eV) which restricts its use to the ultraviolet region. Such radiation is not very abundant in the solar radiation that reaches the Earth, which limits the use of TiO_2_ in solar energy utilization [4]. Therefore, many methods have been applied to extend the light absorption of TiO_2_ [5,6,7,8,9,10], in which photosensitization has shown excellent capability compared with other methods such as metals ion doping.

In the past decades, metalloporphyrins have been used as cytochrome P-450 models for the highly efficient homogeneous or heterogeneous oxidation of organic compounds [11,12,13,14,15,16]. Moreover, they are recognized to be the most promising photosensitizers due to their very strong absorption in the 400–500 nm region (Soret band) and in the 500–700 nm region (Q bands) [17]. Recent years, porphyrins-sensitized TiO_2_ photocatalyst have been used for the degradation of pollutants. Li and co-authorsreported the efficient degradation of 4-nitrophenol (4-NP) under visible light irradiation, in which the complex structural porphyrins was used as photosensitizer [18]. Tetra(4-carboxyphenyl) metalloporphyrins adsorbed on TiO_2_ surface were used as sensitizers for the degradation of atrazine under visible light irradiation [19].

However, in the treatments of methyl orange (MO) azo dye waste, little attention has been paid on the photocatalytic degradation with porphyrins-sensitized TiO_2_ composites as catalyst [20]. In the present work, the photocatalytic degradation of methyl orange catalyzed by the simple structural metalloporphyrins (*meso*-tetraphenylmetalloporphyrins) immobilized on TiO_2_ (Degussa P25) has been investigated. Moreover, NBO charges analysis was performed with density functional theory (DFT) in order to explore the influence of metal ion on the photocatalytic activity.

## 2. Results and Discussion

### 2.1. Characterization of the Photocatalysts

#### 2.1.1. SEM Images

**Figure 1:**
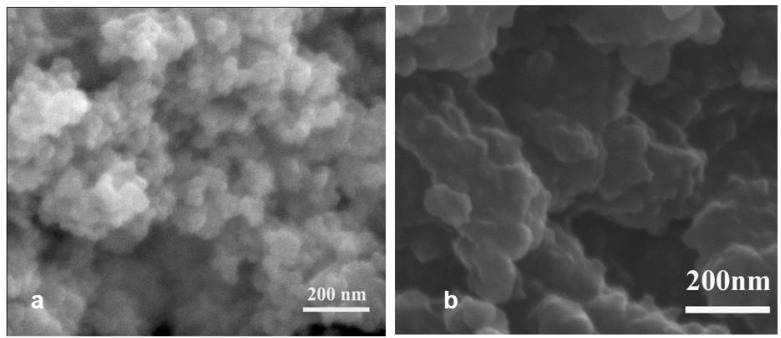
SEM image of TiO_2_ (a) and CuP-TiO_2_ (b).

The morphology of the photocatalysts was examined by scanning electron microscope (SEM). The micrographs of pure TiO_2_ and CuP-TiO_2_ are shown in Figure 1(a) and (b), respectively. Figure 1(a) shows the typical surface of TiO_2_ Degussa P25, with the average crystallite size about 20 nm. When metalloporphyrins was doped on the TiO_2_, aggregates could be observed clearly from the micrographs of CuP-TiO_2_ as shown in Figure 1(b), which indicated the strong adsorption occurred between metalloporphyrins and the TiO_2_ support.

#### 2.1.2. XRD Analysis

**Figure 2:**
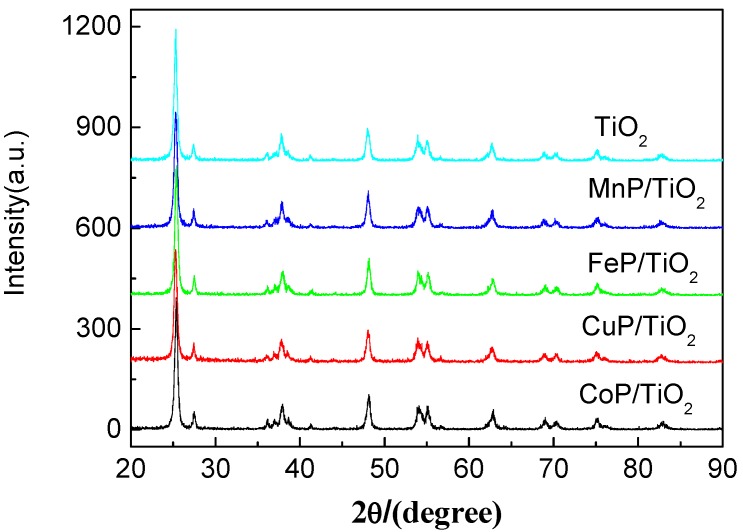
X-ray diffraction patterns of TiO_2_ (P25) and metalloporphyrins-TiO_2_ catalysts.

The crystal phases of TiO_2_ and different metalloporphyrins-TiO_2_ catalysts were analyzed by means of X-ray diffraction, as shown in Figure 2. For TiO_2_ Degussa P25, the peaks at 25.3° and 27.4° are the characteristic reflection for anatase and rutile, respectively [21]. It could be known that the structure of metalloporphyrins-immobilized TiO_2_ are unchanged to those observed for the bare TiO_2_, indicating that the immobilization process did not destroy the characteristic structure of TiO_2_. 

#### 2.1.3. FT-IR spectra

**Figure 3:**
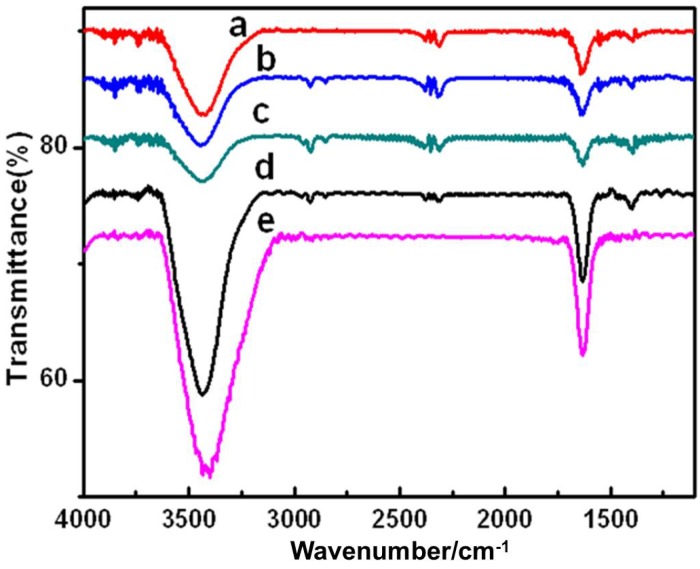
FTIR spectra of TiO_2_ (P25) and metalloporphyrins-TiO_2_ catalysts, (a): CoP-TiO_2_, (b): FeP-TiO_2_, (c): MnP-TiO_2_, (d): CuP-TiO_2_, (e): TiO_2 _(P25).

The bonding characteristics of functional groups in TiO_2_ and different metalloporphyrins-TiO_2_ composites were identified by FT-IR spectroscopy as shown in Figure 3. A broad band at 3425 cm^−1^ is the primary O-H stretching of the hydroxyl functional group. The band around 1630 cm^-1^ is attributed to the bending vibration H-OH groups for TiO_2_ Degussa P25. 

After metalloporphyrins was immobilized on TiO_2_, the basic characteristic peaks for TiO_2_ hardly changed. The stretching vibrations of C-C, C-H bonds can be observed in the supported catalysts, indicating that there contains metalloporphyrins on the surface of TiO_2_. These changes indicated the presence of metalloporphyrins was immobilized onto TiO_2_ Degussa P25. Further observation shows that the peaks corresponding to the stretching vibrations of hydroxyl groups are broader and stronger in TiO_2_ than that of supported catalysts, indicating that the decrease of hydroxyl groups after metalloporphyrins is immobilized onto TiO_2_.

#### 2.1.4. DR UV-vis Spectra

**Figure 4:**
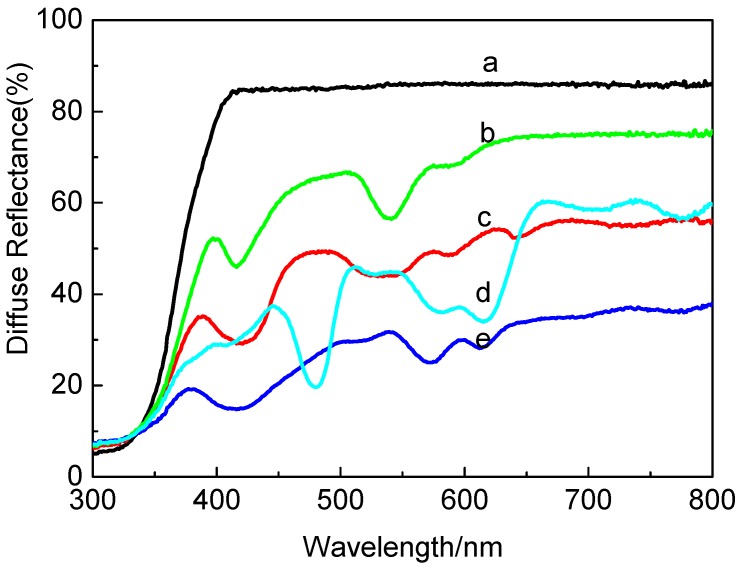
The diffuse reflectance spectra of TiO_2_ (P25) and metalloporphyrins-TiO_2_ catalysts, (a): TiO_2_ (P25), (b): CuP-TiO_2_, (c): CoP-TiO_2_, (d): MnP-TiO_2_, (e): FeP-TiO_2_.

The presence of metalloporphyrins in the TiO_2_ Degussa P25 was determined by UV-Vis diffuse reflectance (UV-Vis-DRS) as shown in Figure 4. The UV-Vis spectra of metalloporphyrins using CH_2_Cl_2_ as a solvent were recorded on a Shimadzu UV-2450 UV-Vis spectrophotometer in the range of 200–800 nm, and all results were summarized in Table 1.

**Table 1:** UV-vis data of metalloporphyrins and DR data of photocatalysts.

Compounds	*λ*_max_（nm）			
FeP	416	513	569	603
FeP-TiO_2_	413	517	587	612
CoP	410	527	580	635
CoP-TiO_2_	412	530	589	642
MnP	407	477	574	609
MnP-TiO_2_	410	479	577	615
CuP	414	538	586	619
CuP-TiO_2_	418	540	590	622

Obviously, there is no absorption above 400 nm for TiO_2_, while the supported catalysts exhibit the characteristic peaks of metalloporphyrins (Table 1), indicating that metalloporphyrins successfully loaded onto the TiO_2_ surface while maintaining the porphyrin framework. It was further observed that the DR spectra of the photocatalysts had a significant red shift compared with the metalloporphyrins in CH_2_Cl_2_ solution, respectively. The red shift could be interpreted as the result of an absorption between the metalloporphyrins and TiO_2_ surface. 

### 2.2. Photocatalytic Activity

**Figure 5:**
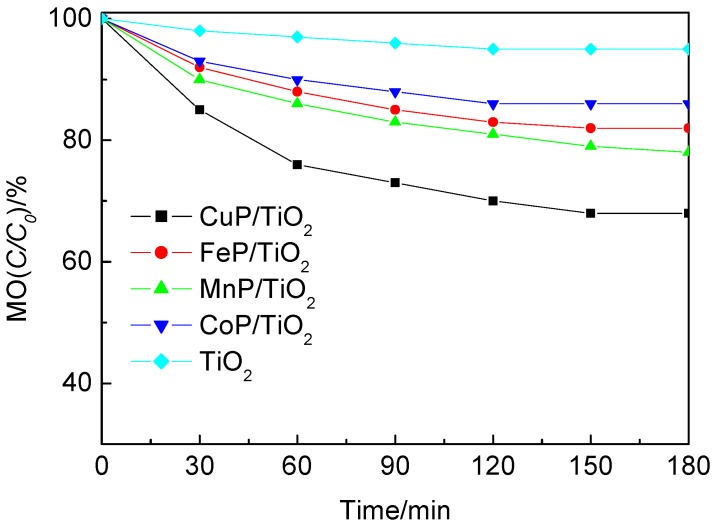
Photodegradation of MO by TiO_2_ and MP-TiO_2_ catalysts under visible light irradiation.

Firstly, the photocatalytic activities of TiO_2_ (P25) and the supported catalysts were measured by the degradation of methyl orange aqueous solution under visible light irradiation. The residual concentration ratios *c*/*c_0_* of MO *versus* degradation time (t) was shown in Figure 5. 

In Figure 5, *c_0_* is the initial concentration of MO, and *c* is the concentration of MO after visible light irradiation. As shown in Figure 5, TiO_2_ presented poor activity for the degradation of MO under visible light irradiation, which the degradation ratio was less than 10% even the irradiation time reached up to 180 min. It could be seen that all MP-TiO_2_ (M= Fe, Co, Mn and Cu) showed higher photocatalytic activity than the bare TiO_2_ Degussa P25. Among these MP-TiO_2_ catalysts, CuP-TiO_2_ exhibited the best efficiency for the degradation of MO. The efficiency of the supported catalysts was found to follow the order: CuP-TiO_2_ > MnP-TiO_2_ > FeP-TiO_2_ > CoP-TiO_2_. 

**Figure 6:**
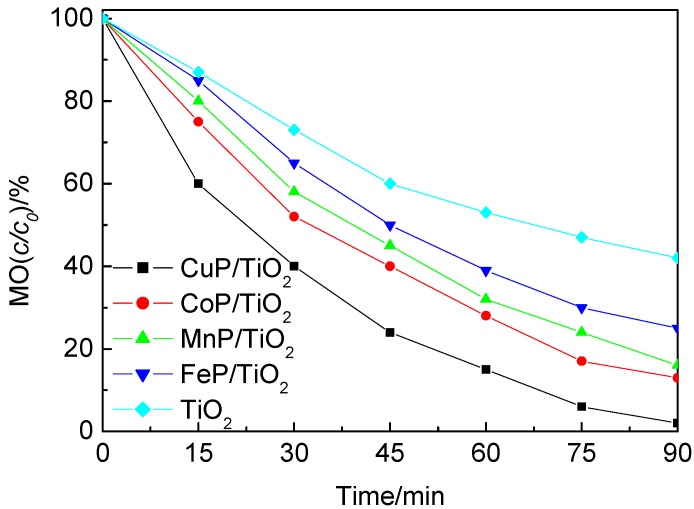
Photodegradation of MO by TiO_2_ and MP-TiO_2_ catalysts under ultraviolet light irradiation.

When the degradation reaction of MO was conducted under ultraviolet light irradiation, the photocatalytic efficiencies of all samples were extremely enhanced compared with those observed under visible light conditions and the results were shown in Figure 6. The results indicate that the activities of MP-TiO_2_ are higher than those of TiO_2_. Compared with other supported catalysts, CuP-TiO_2_ also exhibited the best efficiency for the degradation of MO under UV light irradiation, which the degradation ratio reached up to 98% after 1.5 h of irradiation. The efficiency of the catalysts was found to follow the order: CuP-TiO_2 _> CoP-TiO_2 _> MnP-TiO_2 _> FeP-TiO_2_. These results indicate that metal ion plays an important role in the photoreactivity. With the partially filled *d* orbitals, Cu (II) ions are capable of fluorescence quenching by electron or energy transfer [22]. In addition, according to the diffuse reflectance spectra as listed in Table 1, CuP-TiO_2_ has more adsorption compared with other metalloporphyrins supported on TiO_2_ composite catalysts, especially in the longer wavelength, which is also responsible for the high efficiency of Cu-TiO_2_.

As discussed above, TiO_2_ Degussa P25 impregnated with metalloporphyrins were more efficient catalysts compared to bare TiO_2_ for the photodegradation of MO no matter whether under visible or ultraviolet light irradiation. Metalloporphyrins attached to TiO_2_ could facilitate the charge transfer between the TiO_2_ valence band and the methyl orange molecules. The metal ions may adsorb the MO molecules and encourage the charge-transfer process. 

According to previous report [23,24,25,26], the process of CuP-TiO_2_ photocatalytic reaction can be intitiated by the excitation of the ground state of the sensitizer (CuP) *via* one photon transition (*hv*) to its singlet excited state ^1^[CuP]*. Triplet state ^3^[CuP]* of the sensitizer could be generated by a process of intersystem crossing (equation 1). Both excited states can produce ·O_2_^-^ with molecular oxygen under irradiation (equation 2). Following a series of reactions produce reactive intermediate such as ·OH could also be generated (equation 3). 


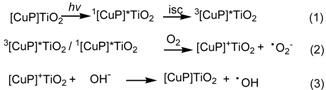


**Figure 7:**
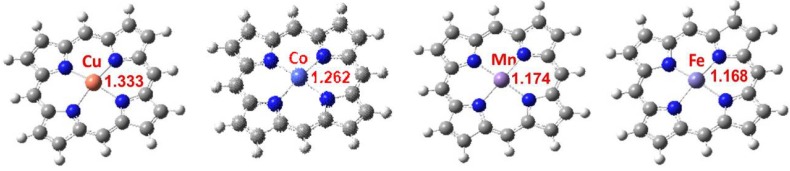
The optimized molecular structures of the metalloporphyrins with different metal ion.

To get a better understanding for the influence of metal ion on the photocatalytic activity, calculations were performed with the Gaussian03W package using the density functional theory (DFT) [27]. Full geometry optimizations of different metalloporphyrins were performed employing Becke’s three parameter Lee-Yang-Parr correlation functions (B3LYP) combined with 6-31G basis set. The optimized structures of MP-TiO_2_ (M= Fe, Co, Mn and Cu) are presented in Figure 7.

According to the calculated results, the NBO charge of metal ion for CuP, CoP, FeP and MnP are 1.333, 1.262, 1.174 and 1.168 eV, respectively. It is well known that methyl orange is an anionic dye. Therefore, the interaction between positively charged surface of the supported catalysts and MO ion could happen.

Due to the higher positive NBO charge of CuP, the methyl orange ion is easily adsorbed by CuP-TiO_2_ catalyst due to the increase of induced interaction, resulting in higher photocatalytic activity. In addition, for the metalloporphyrins with different metal ions, the order of activity under ultraviolet light irradiation is exactly consistent with the calculated NBO charge orders of the metal ions, which indicates the interaction between MO and catalyst is the key step for the photocatalytic degradation.

## 3. Experimental

### 3.1. Preparation of the Metalloporphyrins

All chemical reagents were of analytical grade and were used without further purification unless indicated. Pyrrole was redistilled before use. Metalloporphyrins catalysts were prepared according to the previous procedures [28]. The structures of catalysts were confirmed by elementary analysis, IR and UV-Vis spectra. FT-IR spectra were obtained on a Bruker 550 spectrometer. UV-Vis spectra were recorded on a Shimadzu UV-2450 UV-Vis spectrophotometer. Elemental analysis data were obtained on Vario EL III.

### 3.2. Preparation of the Photocatalysts

In this work, TiO_2_ Degussa P25 (75% anatase and 25% rutile form, surface area 50 ± 15 m^2^/g) was used for the immobilization of the simple structural metalloporphyrins (Fe, Co, Mn and Cu). The loaded samples used as photocatalyst for the degradation experiments were prepared in the following way: metalloporphyrins (12 μmol) was dissolved in CH_2_Cl_2_ (30 mL) and TiO_2_ (P25, 2 g) was added to this solution. The resulting suspension was magnetically stirred at room temperature for 10 h. The catalyst was collected by removing solvent under vacuum and dried at 60 °C for 12 h. The photocatalyst was marked as FeP-TiO_2_, CoP-TiO_2_, MnP-TiO_2_ and CuP-TiO_2_, respectively.

The surface morphology and compostion of the samples were investigated by scanning electron microscopy (Quanta 400F) with energy dispersive X-ray (EDX) spectroscopy. The crystalline structure of the samples was determined by an X-ray diffraction (XRD) using a Rigaku D/MAX-RB diffractometer with Cu Kα source and operated at 40 kV and 40 mA. The diffraction patterns were recorded in the 2θ value range of 20–90° with a scanning rate of 10 °/min. UV-Vis spectra were recorded on a Shimadzu UV-2450 UV-Vis spectrophotometer. FT-IR spectra were obtained on a Bruker 550 spectrometer.

### 3.3. Photocatalytic Activity

The photocatalytic activity of the catalysts was evaluted by the photocatalytic degradation of MO in aqueous solution. The photocatalysis experiments were performed in a batch reactor using a 500 W high-pressure Hg lamp as light source (Shanghai Bilang Co. Ltd), which was positioned 10 cm away from the batch reactor. A circulating water jacket was used to cool the batch reactor and the temperature was kept at around 25 °C. A 400 nm cut-off filter was used under the visible irradiation.

In a typical experiment, the reaction suspension consisting of MO aquesous solution (20 mg L^−1^, 150 mL) and catalyst (0.04 g) was stirred with a magnetic bar. Dioxygen was bubbled into the suspension. In all cases, the mixture was kept in the dark for 30 min to ensure that the adsorption-desorption equilibrium was reached before irradiation. After visible light or ultraviolet light irradiation, the sample was withdrawn from the suspention every 30 min during the irradiation for the determination of the absorbance change of MO. The catalyst was removed by centrifugation and remaining MO concentration in the solution was measured by light absorption of the clear solution at 464 nm.

## 4. Conclusions

In conclusion, *meso*-tetraphenyl metalloporphyrins (Fe, Co, Mn and Cu) adsorbed on TiO_2_ (Degussa P25) photocatalysts were efficient for the degradation of MO aqueous solution under ultraviolet light irradiation. CuP-TiO_2_ showed the most excellent activity for the photodegradation of MO no matter under visible or ultraviolet light irradiation. The DFT calculation results suggest that methyl orange ion is easier to be adsorbed by CuP-TiO_2_ due to the more positive charge of Cu ion. 
